# Cellular Effects of HER3-Specific Affibody Molecules

**DOI:** 10.1371/journal.pone.0040023

**Published:** 2012-06-29

**Authors:** Lovisa Göstring, Magdalena Malm, Ingmarie Höidén-Guthenberg, Fredrik Y. Frejd, Stefan Ståhl, John Löfblom, Lars Gedda

**Affiliations:** 1 Department of Radiology, Oncology and Radiation Science, Biomedical Radiation Sciences, Uppsala University, Uppsala, Sweden; 2 Department of Molecular Biotechnology, School of Biotechnology, Royal Institute of Technology (KTH), AlbaNova University Center, Stockholm, Sweden; 3 Affibody AB, Solna, Sweden; Bioinformatics Institute, Singapore

## Abstract

Recent studies have led to the recognition of the epidermal growth factor receptor HER3 as a key player in cancer, and consequently this receptor has gained increased interest as a target for cancer therapy. We have previously generated several Affibody molecules with subnanomolar affinity for the HER3 receptor. Here, we investigate the effects of two of these HER3-specific Affibody molecules, Z05416 and Z05417, on different HER3-overexpressing cancer cell lines. Using flow cytometry and confocal microscopy, the Affibody molecules were shown to bind to HER3 on three different cell lines. Furthermore, the receptor binding of the natural ligand heregulin (HRG) was blocked by addition of Affibody molecules. In addition, both molecules suppressed HRG-induced HER3 and HER2 phosphorylation in MCF-7 cells, as well as HER3 phosphorylation in constantly HER2-activated SKBR-3 cells. Importantly, Western blot analysis also revealed that HRG-induced downstream signalling through the Ras-MAPK pathway as well as the PI3K-Akt pathway was blocked by the Affibody molecules. Finally, in an *in vitro* proliferation assay, the two Affibody molecules demonstrated complete inhibition of HRG-induced cancer cell growth. Taken together, our findings demonstrate that Z05416 and Z05417 exert an anti-proliferative effect on two breast cancer cell lines by inhibiting HRG-induced phosphorylation of HER3, suggesting that the Affibody molecules are promising candidates for future HER3-targeted cancer therapy.

## Introduction

The Epidermal growth factor receptor (EGFR) family of receptor tyrosine kinases consists of four members: EGFR (ErbB1), HER2 (ErbB2), HER3 (ErbB3) and HER4 (ErbB4). Binding of extracellular growth factors induces receptor homo- or heterodimerisation and activation of the intracellular tyrosine kinase domains, triggering downstream signalling cascades. The signalling eventually leads to proliferation, migration and resistance to apoptosis [1]. Hence, aberrant regulation of the receptor signalling contributes to development of various malignancies such as breast, ovarian, head and neck and lung cancer among others [2]. The most well-characterised receptors of this family are EGFR and HER2, which are both overexpressed in a number of cancer types, respectively. Inhibitors to these two receptors have been developed as cancer therapeutics during the last years, including receptor-specific antibodies and low molecular weight tyrosine kinase inhibitors [3]. Recently, the HER3 receptor has gained interest as a potential new target of cancer therapy [4], [5]. HER3 differs from the other receptor members in that it lacks a fully functional tyrosine kinase domain [6], but it has two natural ligands, heregulin (or neuregulin 1) and neuregulin 2 [7]. Upon ligand binding, HER3 heterodimerises with other receptors of the EGFR family, forming a functional signalling unit. EGFR, HER2 and HER4 are all possible dimerisation partners of HER3, but HER2 and HER3 form a particularly potent heterodimer, which is regarded as an oncogenic unit in many HER2-driven breast cancers [8], [9]. In these cancers, functionality of both HER2 and HER3 has been shown essential to maintain tumour proliferation. HER2 lacks ligands of its own, but is more resistant to internalisation and degradation than the other receptors [10]. HER3, on the other hand, is unique in that it has a number of direct binding sites for the p85 subunit of phosphoinositide-3-kinase (PI3K), which enables more efficient signalling via the PI3K-AKT pathway compared to the other EGFRs [11]. It is considered that downregulation of this signalling pathway, which mediates tumour cell proliferation and survival, is important for anti-proliferative effects of therapeutic agents targeting the epidermal growth factor receptors [12], [13], [14].

Although therapy against EGFR and HER2 has been successful in many cases, patients have a tendency to develop resistance to the inhibitory agents [15]. It has been shown that over-activation of HER3 accounts for some of this resistance, either via increased receptor phosphorylation and cell surface localisation [16], or via overexpression of the receptor or upregulation of the ligands, forming an autocrine loop [17], [18]. Therefore, the HER3 receptor is an interesting target for new antitumour therapeutics and currently two antibodies against HER3, MM-121 (Merrimack Pharmaceuticals) and U3-1287 (AMG888, U3 Pharma GmbH/Daichi Sankyo Inc.), are in clinical trials. It should be noted that the MM-121 anti-HER3 antibody that is under development by Merrimack Pharmaceuticals is formatted as an IgG2 antibody, thus unable to induce significant antibody-dependent cellular cytotoxicity (ADCC) and relies on heregulin (HRG) blocking for therapeutic effect, indicating that non-immunoglobulin based binders may have potential for similar applications. Additionally, a bispecific antibody against both HER2 and HER3, MM-111 (Merrimack Pharmaceuticals), as well as a bispecific antibody against EGFR and HER3 [19], MEHD7945A (Genentech, A member of the Roche group) are being tested in the clinic (for more information see www.clinicaltrials.gov).

We have previously described the selection and affinity maturation of HER3-binding Affibody molecules [20]. Affibody molecules are small three-helix proteins (approximately 6.5 kDa), originally derived from one of the subunits of staphylococcal protein A. Using various display formats such as phage and staphylococcal display, Affibody molecules can be selected against a protein of interest [21], [22], [23]. For instance, display of recombinant Affibody libraries has generated EGFR- and HER2-specific Affibody molecules, which have shown promising results for both tumour imaging and therapeutic applications [24], [25], [26], [27]. Affibody molecules (like some other alternative scaffolds) have complementary properties to antibodies, e.g. i) much smaller size, ii) lack of disulphide bonds and free cysteines, iii) high stability and solubility and iv) efficient production routes using prokaryotic hosts and chemical peptide synthesis are available [22]. The small size results in a much more rapid in vivo biodistribution through efficient extravasation and tissue penetration. The small size also enables flexible engineering, including straightforward construction of bi/multispecific binders, directed site-specific modification for conjugation of e.g. small molecular drugs or chelators for subsequent radiolabelling as well as tailored in vivo half-life through various technologies (e.g. PEGylation and fusion to albumin-binding domains). The rapid biodistribution of Affibody molecules also results in extremely high contrast in in vivo molecular imaging applications, as have previously been demonstrated in numerous publications with binders against for example HER2 and EGFR [22].

In this paper, we have investigated the effects of two affinity-matured HER3-binding Affibody molecules, Z05416 and Z05417, on several cancer cell lines. As previously described [20], these Affibody molecules have high sequence similarity, only differing at 4 out of 58 amino acid positions, and both have subnanomolar affinity (0.8 and 0.7 nM respectively) for HER3. However, it was not known whether they could exert any receptor antagonizing effect on living cells, or what effect the difference in amino acid sequence would have. In the current *in vitro* studies, specific receptor binding on cells was shown using flow cytometry and confocal microscopy. Subsequently, we studied the effects of the HER3-specific Affibody molecules on receptor signalling and cellular growth. We show that HRG-induced phosphorylation of HER3 as well as downstream Akt and Erk-mediated signalling can be suppressed by Z05416 and Z05417. Ultimately, treatment of MCF-7 and SKBR-3 cells with Z05416 or Z05417 was shown to inhibit HRG-induced cell proliferation.

## Materials and Methods

### Affibody molecules

The affinity maturated, HER3-specific Affibody molecules His-Z05416-Cys and His-Z05417-Cys were used in this study and are hereafter referred to as Z05416 and Z05417 respectively [20]. The Affibody molecules were produced in *E. coli* and purified by IMAC as previously described [20]. As a negative control, a Taq-polymerase binding Affibody, His-Z01155-Cys, was used (here referred to as ZTaq). Since Taq polymerase is not naturally occurring in cells, ZTaq is not supposed to bind to or affect cells. To avoid spontaneous dimer formation through disulfide bonding, the C-terminal cysteines of all Affibody molecules were blocked by *N*-ethylmaleimide according to a previously described protocol [20].

### Cell lines

The human cancer cell lines AU565, SKBR-3 and SKOV-3 were obtained from ATCC (Manassas, VA, USA), while MCF-7 cells were obtained from DSMZ (Braunschweig, Germany). All cells were cultured in medium supplemented with foetal calf serum (FCS; 10%), L-glutamine and penicillin/streptomycin. In addition, the RPMI growth medium for MCF-7 cells was supplemented with sodium pyruvate and non-essential amino acids. The RPMI growth medium for AU565 cells (RPMI medium) was also supplemented with D-glucose, sodium bicarbonate, sodium pyruvate and HEPES. Both SKBR-3 and SKOV-3 cells were grown in McCoy's medium.

### Fluorophore-labelling of Affibody molecules

Z05416, Z05417 and ZTaq were labelled with maleimide-coupled fluorophore via their C-terminal cysteine. Each Affibody molecule was diluted in phosphate-buffered saline (PBS) to 100 nM and reduced with 20 mM dithiothreitol (DTT) for 1 h at room temperature. DTT was removed using NAP-5 columns (GE Healthcare, Uppsala, Sweden) equilibrated in PBS, and five-fold molar excess of Alexa Fluor® 488 C5-maleimide (Invitrogen, Carlsbad, CA) dissolved in dimethylsulfoxide (DMSO) was added. After incubation at room temperature for 1 h in dark, unbound fluorophore was removed using a PD-10 column (GE Healthcare) equilibrated with PBS. Degree of labelling and protein concentration was determined using a NanoDrop ND-1000 (Thermo Scientific, Rockford, IL).

### Biotinylation of heregulin

Biotinylation of heregulin (HRG, NRG1-β1/HRG1-β1 ECD domain, R&D Systems, Minneapolis, MN) was performed using biotin-XX sulfo succinimidyl ester, sodium salt (Invitrogen) in 0.1 M NaHCO_3_ (pH 8.5). 50 µg of HRG was mixed with a four-fold molar excess of biotin and incubated for 1 hour at room temperature. Next, glycine (100 mg/ml) was added to stop the labelling reaction prior to removal of excess biotin and buffer exchange using a NAP-5 column (GE Healthcare) equilibrated with PBS. The protein concentration was determined by spectrophotometry.

### Flow cytometry, blocking with unlabelled Affibody molecules

Trypsin treated cells (MCF-7, SKBR-3 and SKOV-3, respectively), 500 000 in each tube, were washed with PBS. The cells were incubated for 15 minutes with PBS or with 15 μM of unlabelled Affibody molecule in PBS to assess specific binding. Corresponding Alexa Fluor® 488-labelled Affibody molecules were then added to a final concentration of 150 nM and incubated for 90 minutes. After washes in PBS, the cells were analysed using a BD LSR II flow cytometer (BD Biosciences, San Jose, CA). All incubations were performed on ice, and a cooled centrifuge and ice-cold PBS were used for the washes.

### Immunofluorescence microscopy

Affibody molecules were used for immunofluorescence staining of the human carcinoma cell lines AU565, MCF-7, SKBR-3 and SKOV-3. As a positive control, a polyclonal goat anti-human HER3 antibody (AF234, R&D systems) was used. All affinity reagents were diluted in PBS supplemented with 4% FCS and washing was performed using ice-cold PBS. Approximately 20 000 cells/well were seeded in a 96-well plate with glass bottom and grown overnight. The next day, cells were washed prior to addition of Affibody molecules or the HER3-specific antibody at a concentration of 1.8 and 0.033 µM respectively. After a 1-hour incubation, cells were washed and a goat anti-Affibody antibody (0.033 µM, Affibody AB, Solna, Sweden) was added to wells previously incubated with Affibody molecules and incubated for 1 hour. Subsequently, all binding events were detected by a final incubation with a chicken anti-goat IgG conjugated with Alexa Fluor® 488 (Invitrogen) at a concentration of 0.013 µM for 1 hour.

In a second experiment, competitional binding between Affibody molecules and HRG to AU565 cells was evaluated. Cells were stained with 100 nM Affibody molecules for 10 minutes prior to addition of 100 nM biotinylated HRG in 100 nM Affibody molecules. After 10 minutes, cells were washed and streptavidin conjugated with Alexa Fluor® 488 (4 µg/ml) was added to each well. As negative controls, HRG was added to cells preincubated with PBS as well as to cells incubated with Z01155. As a negative staining control, streptavidin Alexa Fluor® 488 was added to cells not preincubated with either Affibody molecules or HRG. After completed staining, cells were washed, fixed in paraformaldehyde for 15 minutes followed by nuclear staining with (4′,6-diamidino-2-phenylindole) DAPI for 5 minutes. Finally, 1xPBS diluted in 85% glycerol was added and the wells were covered with foil. Cell staining was analysed using a Leica SP5 confocal laser-scanning microscope with a 63×, 1,4 NA oil immersion objective.

### Cell treatment and lysis

Cells were seeded in 6-well plates (600 000–800 000/well), allowed to grow for 24 hours in complete medium and starved for 24 hours in serum-free medium with 0.1% BSA. Heregulin (0.05 nM; NRG1-β1/HRG1-β1 EGF domain, R&D Systems) and Affibody molecules (100 nM) were diluted in starvation medium and cells were treated in duplicates with combinations of heregulin and Affibody molecules for 10 minutes at 37°C. The cells were washed twice with ice cold PBS, and lysis buffer (1% NP-40, 20 mM Tris (pH 8.0), 137 mM NaCl, 10% glycerol, 2 mM EDTA, 1 mM activated sodium orthovanadate, protease inhibitor cocktail (Sigma, St Louis, MO)) was added to a concentration of 6.7×10^6^ cells per ml lysis buffer. The cells were kept on ice for 30 minutes, detached from the wells with a cell scraper and centrifuged in eppendorf tubes at 14 000 g at 4°C for 15 minutes. The supernatant from each tube was collected and used in the phospho-HER2/3 ELISA and Western blot.

### Phospho-HER2/3 ELISA

ELISA kits from R&D systems (DuoSet® IC) for detection of phosphorylated HER2 and HER3, respectively, were used according to the manufacturer's instructions and as described below. 96-well half area plates were coated with an anti-HER2 or HER3 antibody at 4 µg/ml in PBS at 4°C over night. The plate was washed and blocked with 1% BSA in PBS for 2 hours at room temperature. After washing, 25 µl of “diluent #12” (1% NP-40, 20 mM Tris (pH 8.0), 137 mM NaCl, 10% glycerol, 2 mM EDTA, 1 mM activated sodium orthovanadate) and 25 µl of respective cell lysate was added to each well in duplicates. The plate was incubated for 2 hours, washed and incubated with HRP-labelled anti phospho-tyrosine antibody, diluted 1∶2000 in “diluent #14” (20 mM Tris, 137 mM NaCl, 0.05% Tween 20, 0.1% BSA, pH 7.2–7.4), for 2 hours. The plate was washed and substrate was added (R&D Systems). After 20 minutes, the reaction was stopped with 2 M H_2_SO_4_ and the plate was analysed in an Emax microplate reader (Molecular Devices) at 450 and 570 nm.

### Western Blot

The Western blot was performed as described earlier [28]. In short, the cell lysates (produced as described above) were run on a 3–8% Tris-Acetated gel (NuPAGE, Invitrogen) and transferred to a PVDF membrane (0.45 μm, Immobilon, Millipore, Billerica, MA). The membranes were blocked with 5% BSA in PBS for 2 h and incubated with antibodies (diluted in 1% BSA in PBS) against phospho-Akt (Ser473, Cell Signaling Technology, Beverly, MA), phospho-Erk 1/2 (Thr202/Tyr204, Cell Signaling Technology) and β-actin (Sigma), respectively, at 4°C over night (anti-P-Akt, anti-P-Erk) or at room temperature for 2 h (anti-β-actin). After washes in PBS-T (PBS +0.1% Tween), the membranes were incubated with HRP-anti-mouse (anti-P-Akt, anti-P-Erk) or HRP-anti-rabbit (anti-β-actin) antibodies diluted in 1% BSA in PBS for 1 h at room temperature in the dark. After washes in PBS-T, the membranes were incubated in HRP substrate (Immobilon, Millipore) for 1 minute and detected in a CCD-camera (Fuji, Tokyo, Japan).

### Cell proliferation assays

The human breast cancer cell lines MCF-7 and SKBR-3 were cultured for 5 days in medium supplemented with 2% dialysed FCS (Gibco, Invitrogen) in the presence or absence of Affibody molecules and HRG (NRG1-β1/HRG1-β1 EGF domain, R&D Systems) in order to evaluate the effects of these reagents on cell growth. Cells were seeded in a 96-well plate and the number of cells per well was 1500 for MCF-7 cells and 3000 for SKBR-3, unless stated otherwise. After incubation, the amount of living cells in each well was determined using cell counting kit-8 (CCK-8, Fluka, Sigma Aldrich) according to the manufacturer's recommendations and absorbance was measured at 450 nm using a microplate reader.

The effect of HRG on cells was evaluated by stimulating MCF-7 cells and SKBR-3 by the addition of a dilution series of ten different concentrations of HRG (ranging from 1.5 pM to 10 nM) in 5 replicates.

In a second experiment, Affibody molecules were added to 1500 MCF-7 cells/well or 2000 SKBR-3 cells/well as a 3-fold dilution series of 10 different concentrations ranging from 20 pM to 400 nM for MCF-7 cells, or 40 pM to 800 nM for SKBR-3 cells. Each well was also supplemented with 40 pM HRG. Samples were analysed in four replicates and the obtained data was fitted by nonlinear regression to a sigmoidal dose response curve (variable slope) to obtain IC50 values.

In a final setup, cells were incubated in growth medium supplemented with HRG or Affibody molecules at a final concentration of 40 pM and 40 nM respectively. Each sample was analysed in 5 replicates and results were compared to unstimulated samples (without added HRG or Affibody molecules). Additionally, mean background absorption by the medium alone was subtracted from all data.

## Results

### Flow cytometry

**Figure 1:**
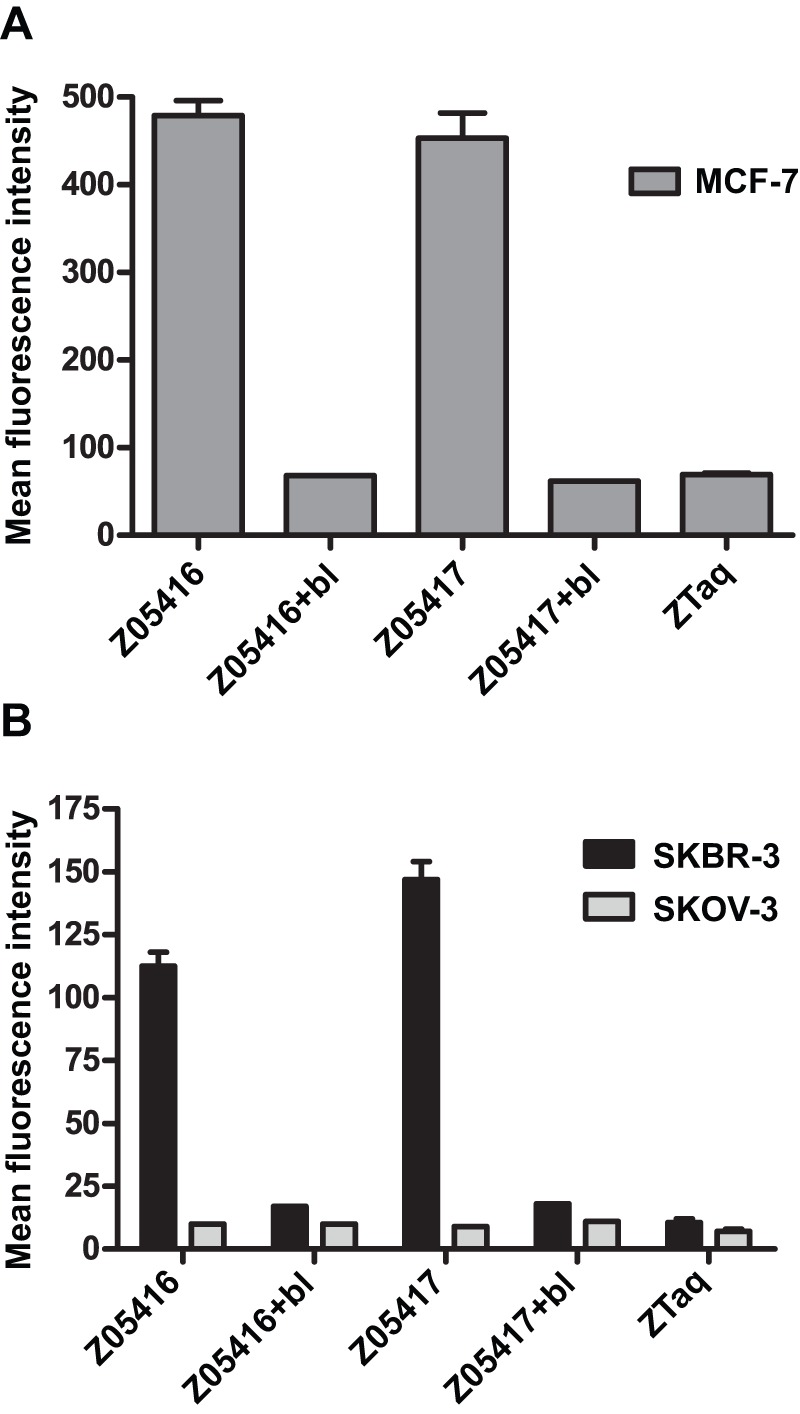
Cell binding of HER3-specific Affibody molecules analysed by flow cytometry. Binding of Alexa Fluor® 488-labelled Affibody molecules (150 nM) to: A. MCF-7, B. SKBR-3 and the HER3-negative cell line SKOV-3. “Bl”  =  blocking with 15 μM of the corresponding, non-labelled Affibody. MCF-7 cells were stained in a separate experiment, whereas SKBR-3 and SKOV-3 were stained simultaneously.

We have previously generated a panel of HER3-specific Affibody molecules with subnanomolar affinity to recombinant HER3 [20]. In order to investigate whether two of the Affibody molecules, exhibiting the highest affinity to the recombinant receptor in biosensor assays, could bind to native HER3 expressed on human cancer cell lines, we performed a flow-cytometric analysis. The results showed that the fluorescently labelled HER3-specific Affibody molecules (Z05416 and Z05417) bound to the HER3-positive breast cancer cell lines MCF-7 and SKBR-3, but not to the HER3-negative/HER2-positive SKOV-3 cell line (Fig. 1A–B). Furthermore, to assess the specificity of the interaction, cells were pre-incubated with 100-fold excess of corresponding, unlabelled Affibody molecules, resulting in complete blocking of the signal from the fluorescent binders. As a negative control, an Affibody molecule (ZTaq) with affinity for *Taq* polymerase was included in the assay. ZTaq did not bind to any of the cell lines, hence supporting the findings that the new HER3 binders interacted specifically with HER3 receptors on these different cell lines.

### Immunofluorescence microscopy

**Figure 2:**
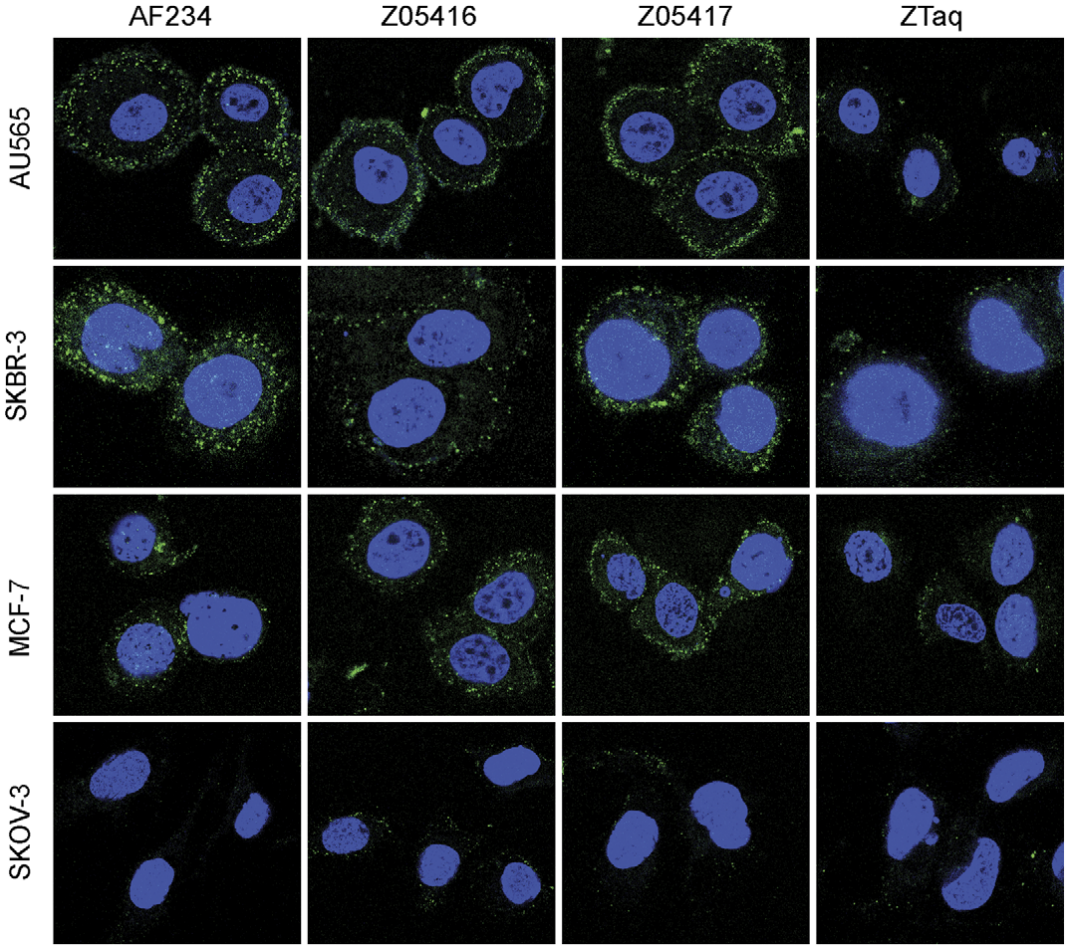
Immunofluorescent staining of human cancer cell lines. Images showing AU565, SKBR-3, MCF-7 and SKOV-3 cells stained with HER3-specific Affibody molecules Z05416 and Z05417, respectively. The polyclonal anti-HER3 antibody A234 and ZTaq were used as positive and negative staining controls, respectively. Affibody molecules and antibodies binding to cells are shown in green while nuclear staining by DAPI is given in blue. AU565 and SKOV-3 images were acquired on the same day using the same detection gain and laser power, enabling comparison between staining intensities. Cell staining of MCF-7 and SKBR-3 was analysed on different days, using different detection gains for optimal image acquisition. Additionally, MCF-7 images were acquired using increased laser power.

To verify the results from the flow-cytometric analysis, cell binding of the HER3-specific Affibody molecules was investigated by immunofluorescent staining of four human cancer cell lines and subsequent confocal microscopy imaging. In addition to the MCF-7 and SKBR-3 cells that were analysed in the flow cytometry assay, the HER3-positive breast cancer cell line AU565 and HER3-negative ovarian cancer cell line SKOV-3 was included. A HER3-specific antibody was used as a positive control. The images of the HER3-positive cells incubated with both Affibody molecules, respectively, revealed a similar staining as for the antibody, with a speckled pattern mainly localised to the cell membrane (Fig. 2). In contrast, the analysis of the HER3-negative and HER2-positive cell line (SKOV-3), as well as the analysis of all cells incubated with the negative control Affibody molecule (ZTaq), showed only weak background fluorescence, further confirming that the cell staining was HER3-specific (Fig. 2).

### Blocking of HRG binding to HER3-overexpressing cells

**Figure 3:**
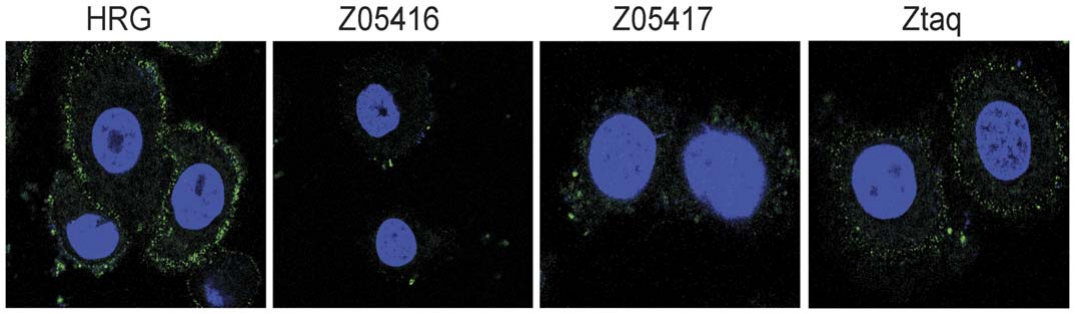
Competitional binding between the natural ligand HRG and Affibody molecules to the HER3-positive breast cancer cell line AU565 visualised by confocal microscopy. AU565 cells were pretreated with Affibody molecules (Z05416, Z05417 or ZTaq) or PBS only (HRG) before addition of HRG in conjugation with a fluorophore. HRG binding to cells is shown in green while nuclear staining by DAPI is shown in blue.

To investigate if the Affibody molecules were able to block the interaction of the natural ligand heregulin with HER3-overexpressing cells and hence potentially inhibit signalling, a competition assay was conducted using confocal microscopy. HER3-positive AU565 cells were preincubated with unlabelled Affibody molecules prior to addition of equimolar amounts of fluorescently labelled HRG. Using confocal microscopy, images were obtained for cells preincubated with Affibody molecules as well as for cells that were only incubated with labelled HRG. The results showed that first of all, addition of labelled HRG resulted mainly in cell membrane staining of the HER3-positive cells and second, preincubation with unlabelled Affibody molecules dramatically decreased the fluorescence from the HRG-binding (Fig. 3). Preincubation with the negative control Affibody molecule (ZTaq) had no significant effect on the HRG-binding, demonstrating that the HER3-specific Affibody molecules are able to bind to the HER3 receptor on the cell surface and thereby block the binding of the natural ligand HRG (Fig. 3). Incubation of cells with only streptavidin conjugated with Alexa Fluor® 488 did not give any cellular staining (data not shown).

### Receptor phosphorylation, ELISA

**Figure 4:**
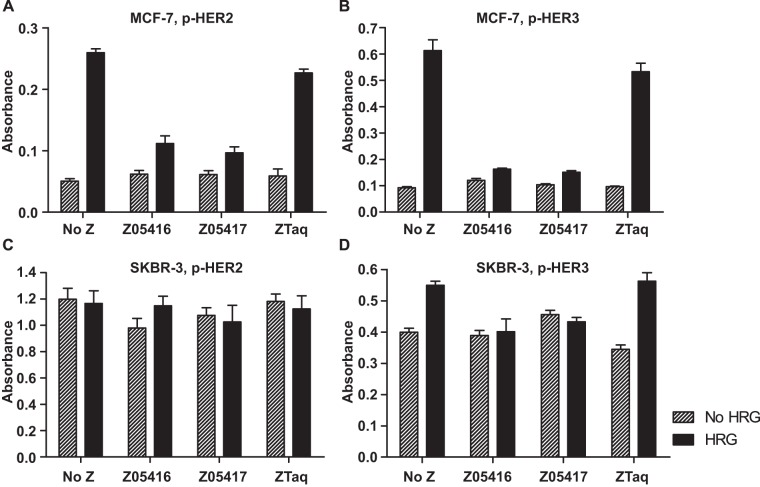
Analysis of receptor phosphorylation of MCF-7 and SKBR-3 cells. Histograms showing ELISA absorbance results for detection of: A. Phospho-HER2 in MCF-7 cells, B. Phospho-HER3 in MCF-7 cells, C. Phospho-HER2 in SKBR-3 cells and D. Phospho-HER3 in SKBR-3 cells. Cells were incubated without HRG (grey bars) or with 0.05 nM HRG (black bars), in combination with the Affibody molecules (100 nM) before being lysed and analysed in an ELISA.

Epidermal growth factor receptors signal through phosphorylation of their intracellular domains as a result of extracellular receptor dimerisation. Since our new HER3-specific Affibody molecules were shown to block the binding of HRG to the HER3 receptor and thereby potentially inhibit receptor activation, we wanted to investigate if binding of the Affibody molecules could also have an effect on receptor phosphorylation. The level of phosphorylation of both the HER2 and the HER3 receptors was analysed in a sandwich ELISA using phosphotyrosine-specific detection antibodies. Two HER3-overexpressing cell lines (MCF-7 and SKBR-3) were used in the assay. Although the cell lines have similar HER3 expression levels, expression of HER2 is around 100-fold higher on SKBR-3 cells. As expected, stimulating both cell lines with the addition of HRG resulted in a significant increase in HER3 tyrosine phosphorylation levels (Fig. 4B and 4D). Notably, the basal level of HER3-phosphorylation was considerably higher for SKBR-3 cells compared to MCF-7 cells. Interestingly, when treated with 100 nM of either HER3-specific Affibody molecule, the HRG-induced phosphorylation was clearly reduced towards the same level as for unstimulated cells (Fig. 4B and 4D). The same pattern was seen in the case of HER2 phosphorylation of MCF-7 cells, although the blocking effect by the HER3-specific Affibody molecules was less pronounced (Fig. 4A). In SKBR-3 cells, the HER2 phosphorylation level was, in contrast, constantly high and not significantly affected by HRG or the Affibody molecules (Fig. 4C), potentially due to the fact that SKBR-3 cells express around 100-fold more HER2 receptor compared to MCF-7. As expected, the negative control ZTaq Affibody molecule did not affect HER3 or HER2 phosphorylation (Fig. 4).

### Akt and Erk phosphorylation, Western blot

**Figure 5:**
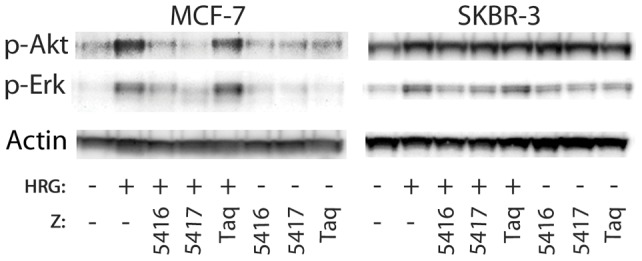
Western blot analysis of phosphorylated Akt and Erk upon addition of heregulin and/or HER3-specific Affibody molecules. Phospho-Akt and phospho-Erk detected by western blot of cell lysates from MCF-7 and SKBR-3 cells treated with (+) or without (−) 0.05 nM heregulin (HRG) and 100 nM Affibody molecules (Z). As a control, β-actin was detected to show that the protein concentrations of the different lysates were equivalent.

The two most important downstream signalling pathways for the epidermal growth factor receptors are the Ras-MAPK and PI3K-Akt pathways. To investigate potential effects of the HER3-specific Affibody molecules on these pathways, two proteins involved in the signalling were studied using Western blot: Erk in the Ras-MAPK pathway and Akt in the PI3K-Akt pathway. As in the receptor phosphorylation assay, the two HER3-positive cell lines MCF-7 and SKBR-3 were included in the analysis. In MCF-7 cells, both Akt and Erk were shown to be activated by 0.05 nM HRG, and more interestingly, this activation could be blocked by addition of the HER3-specific Affibody molecules (Fig. 5). The results were however slightly different for the SKBR-3 cell line. Addition of HRG resulted only in phosphorylation of Erk whereas the phosphorylation of Akt was high even without HRG and no significant effect could be detected upon HRG stimulation (Fig. 5). These results are in line with the observed pattern from the receptor phosphorylation assay, where SKBR-3 cells demonstrated a constantly high activation of HER2 with only a minor response to HRG treatment, probably again due to the high surface expression levels of HER2 on SKBR-3 cells compared to MCF-7. The HER3-specific Affibody molecules could block the HRG-induced phosphorylation of Erk also in SKBR-3, however no effect was seen for the constantly activated Akt (Fig. 5). Furthermore, adding the negative control Affibody molecule (ZTaq) did not result in any detectable decrease in phosphorylation in the assays, supporting the findings that the HER3-specific binding and particularly blocking of HRG is causing the effect on Akt and Erk (Fig. 5). Importantly, even though the Affibody molecules are binding to an epitope on HER3 that is probably overlapping with the epitope of HRG, neither binder demonstrated any detectable agonistic effect on Akt or Erk (Fig. 5). Actin staining showed that all lysates contained similar amounts of protein (Fig. 5).

### Cell proliferation assays

**Figure 6:**
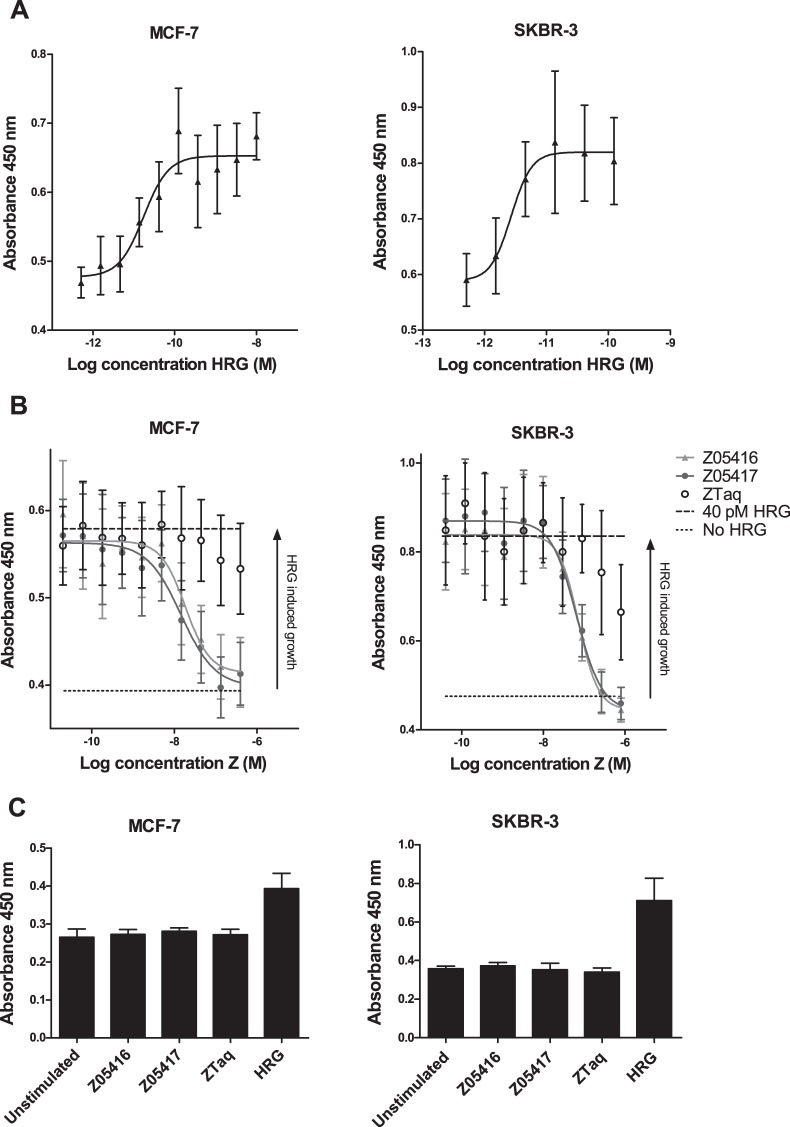
Analysis of cellular growth inhibitory effects of the HER3-specific Affibody molecules. Mean absorbance values at 450 nm ± SD, which is proportional to the number of living cells, is given on the y-axis. A. Proliferation of MCF-7 and SKBR-3 cells grown in a dilution series of HRG. B. Proliferation of MCF-7 and SKBR-3 cells grown in 40 pM HRG and a dilution series of Affibody molecules Z05416, Z05417 or ZTaq. C. Proliferation of cells grown in medium containing 40 nM Affibody molecules, 0.04 nM HRG. Results are compared to unstimulated cells (no Affibody molecules or HRG added).

The observed reduction of HRG-induced HER3 and HER2 receptor phosphorylation as well as the effect on the downstream signalling proteins Akt and Erk, motivated the investigation of potential growth inhibitory effects of Z05416 and Z05417. However, prior to treatment of the cell lines with Affibody molecules, we verified potential growth stimulatory effect of HRG on MCF-7 and SKBR-3 cells in a 5-day proliferation assay. In the assay, HRG was shown to induce growth of both MCF-7 and SKBR-3 cells in a concentration-dependent manner (Fig. 6A). Interestingly, for SKBR-3 cells, the ligand demonstrated an anti-proliferative effect at concentrations of HRG above approximately 0.12 nM (Fig. S1). These results are consistent with previous findings, where HRG have been demonstrated to have a concentration-dependent, biphasic stimulatory/anti-proliferative effect on HER2-overexpressing breast cancer cell lines [29], [30], [31]. The EC50 values obtained here were 17 pM for MCF-7 cells and 2.7 pM for SKBR-3 cells (for SKBR-3 cells the highest HRG concentrations were disregarded in the fitting).

In order to further evaluate the effect of HER3-specific Affibody molecules on the HRG-induced growth of MCF-7 and SKBR-3, cells were cultivated in a fixed concentration of HRG and a dilution series of Affibody molecules for 5 days. Determination of the cell concentration after the 5-day period revealed that the addition of either Z05416 or Z05417 reduced the HRG-induced growth of both MCF-7 and SKBR-3 cells in a concentration-dependent manner and completely blocked the growth-promoting effects of HRG at high Affibody concentrations (Fig. 6B). The obtained IC50 values for Z05416 and Z05417 were 19 and 15 nM respectively for the MCF-7 cell line, and 73 nM and 65 nM respectively for SKBR-3 cells. The results from the phosphorylation studies of Erk and Akt (Fig. 5), suggest that the Affibody-mediated anti-proliferative effect that is observed for SKBR-3 cells is mainly the result of disrupting the Ras-MAPK pathway, potentially due to inhibition of HER3-EGFR dimer formation. Additionally, micromolar concentrations of ZTaq resulted in a slight decrease in proliferation, potentially due to non-specific interactions at high concentrations (Fig. 6B).

Finally, to further investigate the mechanisms behind the growth inhibitory effect of the HER3-specific binders, the two cancer cell lines were treated with Affibody molecules without simultaneous stimulation with HRG. Incubation of cells with 40 nM of Affibody molecules alone showed no significant effect on cellular growth for either cell line, hence supporting the findings that the Affibody molecules affect the growth rate of the HER3-overexpressing cells by binding to HER3 and thus blocking HRG-induced proliferation (Fig. 6C).

## Discussion

The recognition of HER3 as a co-inducer of tumour development, as well as its importance in the context of resistance against HER2 or EGFR-targeted therapies, has lead to increased efforts in finding a way to block the activity of this receptor. In this work, we show that two HER3-specific Affibody molecules, Z05416 and Z05417, are able to block HRG-induced proliferation of the HER3-overexpressing breast cancer cell lines MCF-7 and SKBR-3 *in vitro*. In the proliferation assays, there were no Affibody-mediated effects in the absence of HRG, and altogether these observations suggest that Z05416 and Z05417 exert an anti-proliferative effect on cells due to their ability to block the interaction between HER3 and HRG.

These results are promising for future therapeutic targeting of cancers dependent on the HRG-HER3 signalling pathway. Blocking of ligand-induced signalling of HER3 has previously been proven a successful approach for inhibition of tumour cell growth in *in vitro* studies of several different cancers. For instance, the monoclonal antibody pertuzumab inhibits HER2-HER3 heterodimerisation and hence reduces HRG-induced proliferation of both breast and prostate cancer cells *in vitro* and *in vivo* [32]. Additionally, disruption of signalling through a HRG/HER3 autocrine loop in ovarian cancer cells, by RNAi targeting either HRG or HER3, has been shown to successfully reduce proliferation of the investigated cell lines [33].

The cellular mechanism by which the HER3-specific Affibody molecules affect proliferation can be explained by the *in vitro* phosphorylation patterns of HER3 and HER2. Here, the competition between the HER3-specific Affibody molecules and HRG for HER3 binding was shown to suppress HRG-induced phosphorylation (activation) of HER3 in both MCF-7 cells and SKBR-3 cells. In MCF-7 cells, HER2 phosphorylation was induced by HRG as well and blocked by Z05416 and Z05417, but in SKBR-3 cells this receptor was constantly active and not affected by either HRG or the Affibody molecules. SKBR-3 is known to express extreme amounts of HER2, a feature often linked to constantly activated receptors [34]. MCF-7 and SKBR-3 are both considered to overexpress HER3, but this generally means lower amounts of receptors (40 000–50 000) in comparison to EGFR and HER2-overexpressing cells which may have more than 10^6^ receptors per cell. According to Aguilar et al, the amount of HER2 per cells is reported to be approximately 2×10^6^ in SKBR-3, whereas in MCF-7 it is only 1.5×10^4^. The HER3 density is 2.5×10^4^/cell in MCF-7 and 1.3×10^4^ in SKBR-3 [35].

Two main routes of signalling downstream of HER2 and HER3 are the Ras-MAPK and the PI3K-Akt pathways, which were both investigated via detection of phosphorylated Akt and Erk (MAPK). Since HER3 signals mainly via the PI3K-Akt due to its many PI3K binding sites, this pathway was expected to be most affected by HRG treatment and Affibody-mediated HER3 blocking. However, both Akt and Erk were phosphorylated by HRG treatment in MCF-7 cells, and this phosphorylation was inhibited by Z05416 and Z05417. A similar pattern was seen for Erk in SKBR-3 cells, but in these cells Akt was phosphorylated already in unstimulated cells. This has also been reported by other groups and is believed to be the result of ligand-independent receptor dimerisation due to HER2 overexpression [13], [36]. Thus, our results show that HRG-induced SKBR-3 proliferation may be inhibited by the HER3-binding Affibody molecules without inactivation of the PI3K-Akt pathway.

Specific HER3 binding of Z05416 and Z05417 was demonstrated using both flow cytometry and fluorescence microscopy assays. The moderate intensity of the signals was in concordance with the relatively low amounts of HER3 on the investigated cell lines, as discussed above. With this in mind, high-affinity binders are probably required for successful tumour imaging. The molecules investigated in this study might therefore be suitable for potential therapeutic approaches in cancer. Taken together, the results presented here are promising, and support additional studies of the effect of Z05416 and Z05417 on tumour growth in xenografted mice, to further evaluate the therapeutic potential of these Affibody molecules *in vivo*. In the future, HER3-specific Affibody molecules may be useful in treatment of cancers where the HER3/HRG signalling is involved, potentially in combination with EGFR or HER2-targeted therapies. An interesting field in the context of dual tumour targeting is the generation of bispecific affinity proteins, an approach proven suitable for Affibody molecules [37]. Evaluation of bispecific Affibody molecules targeting HER3 in combination with other receptors of the HER-family would be of great interest due to the complex signalling of these receptors and the importance of HER3 in the context of cancer. To summarize, two previously developed HER3-specific Affibody molecules, with subnanomolar affinity for recombinant HER3, were investigated in this study in terms of potential effects on HER3-positive cancer cell lines in vitro. The results demonstrated that the new binders recognized native HER3 on several cancer cell lines, blocked the natural ligand heregulin from binding to the receptor on the cell surface, decreased the phosphorylation of HER2 and HER3 as well as the downstream signalling proteins Erk and Akt, and finally, inhibited the heregulin-induced proliferation of two HER3-positive cancer cell lines.

## Supporting Information

Figure S1 **Analysis of SKBR-3 cell proliferation in presence of HRG.** Mean absorbance values at 450 nm ± SD, which is proportional to the number of living cells, is given on the y-axis. Proliferation of SKBR-3 cells grown in a dilution series of HRG. The bell-shaped curve demonstrates a biphasic effect of HRG on cell growth in a concentration dependent manner.
(EPS)
